# Machine learning-based risk prediction models for type 2 diabetes in primary care: a scoping review

**DOI:** 10.3389/fpubh.2026.1946070

**Published:** 2026-09-09

**Authors:** Rashita Ravi, Jeby Jose Olickal

**Affiliations:** Department of Public Health, Amrita Institute of Medical Sciences, Amrita Vishwa Vidyapeetham, Kochi, Kerala, India

**Keywords:** artificial intelligence, machine learning, primary care, risk prediction, type 2 diabetes mellitus

## Abstract

**Background:**

Type 2 diabetes mellitus (T2DM) is a major global public health challenge, with many individuals remaining undiagnosed until complications develop. Machine learning (ML)-based risk prediction models have the potential to support early identification of individuals at increased risk using primary care data. However, the characteristics and applicability of these models within primary care settings have not been comprehensively mapped.

**Objective:**

To systematically map the available evidence on machine learning (ML)-based models for risk prediction, early detection, and case-finding of type 2 diabetes in primary care, and to summarize their characteristics, including predictors, modeling approaches, validation strategies, and model performance.

**Methods:**

A scoping review was conducted following the Joanna Briggs Institute methodology and reported according to the Preferred Reporting Items for Systematic Reviews and Meta-Analyses extension for Scoping Reviews (PRISMA-ScR). PubMed/MEDLINE, Scopus, and Ovid MEDLINE were searched for English-language studies published between January 2011 and December 2025. Primary research describing ML-based risk prediction models developed, validated, evaluated, or intended for implementation in primary care was eligible. Data were extracted using a structured charting form and synthesized descriptively.

**Results:**

The search identified 186 records, of which four studies met the inclusion criteria. The included studies were conducted in Sweden, Canada, Saudi Arabia, and Hong Kong between 2024 and 2025. Three studies focused on model development and internal validation, while one externally validated previously developed non-laboratory prediction models. A range of ML approaches was identified, including stochastic gradient boosting, federated learning, multilayer perceptron, random forest, support vector classification, naïve Bayes, and decision tree algorithms, with logistic regression commonly used as a comparator. Models primarily utilized routinely collected demographic, anthropometric, lifestyle, and electronic health record-derived variables. Most studies reported moderate-to-good predictive performance; however, evidence regarding external validation, calibration, and prospective implementation within routine primary care remained limited.

**Conclusion:**

Evidence on ML-based risk prediction models for T2DM applicable to primary care remains limited despite growing interest in AI for diabetes prediction. Existing models demonstrate promising predictive performance using routinely available clinical information, but greater emphasis is needed on external validation, calibration, prospective implementation, and evaluation across diverse primary care populations before widespread clinical adoption.

**Review registration:**

Open Science Framework https://osf.io/mbfrz.

## Introduction

Type 2 diabetes mellitus (T2DM) is one of the fastest-growing non-communicable diseases and remains a major global public health challenge. According to the International Diabetes Federation (IDF), an estimated 537 million adults were living with diabetes worldwide in 2021, and this number is projected to increase to 783 million by 2045, with the greatest increases expected in low- and middle-income countries (1). Despite advances in diabetes care, nearly half of all individuals with diabetes remain undiagnosed, delaying timely diagnosis and increasing the risk of long-term complications, including cardiovascular disease, nephropathy, neuropathy, retinopathy, and premature mortality (2, 3). These trends underscore the need for effective strategies to identify individuals at high risk of T2DM before disease onset, enabling timely preventive interventions and reducing the long-term burden of diabetes.

Early identification of individuals at high risk of T2DM is fundamental to diabetes prevention, as timely interventions can delay or prevent disease onset and its associated complications (4). In primary care, screening for diabetes and prediabetes primarily relies on laboratory-based investigations, including fasting plasma glucose (FPG), oral glucose tolerance testing (OGTT), and glycated hemoglobin (HbA1c), which are recommended diagnostic tests in current clinical guidelines (5). Although these investigations demonstrate high diagnostic accuracy, their routine implementation for opportunistic or population-level screening may be constrained by the need for laboratory infrastructure, trained personnel, repeated patient visits, and associated costs, particularly in resource-constrained settings (6, 7). In addition, increasing patient volumes and competing clinical priorities may further limit opportunities for systematic risk assessment in primary care. Consequently, many individuals at increased risk remain unidentified until biochemical abnormalities become apparent, reducing opportunities for timely preventive interventions. These challenges highlight the need for practical, scalable, and non-invasive approaches that can identify individuals at high risk of T2DM using routinely available clinical information before confirmatory laboratory testing is required.

Artificial intelligence (AI) is a broad field that enables computers to perform tasks that typically require human intelligence. Machine learning (ML), a subset of AI, uses algorithms that learn patterns from data to make predictions or decisions. Recent advances in ML have created new opportunities for improving T2DM risk prediction in healthcare (8–10). Unlike conventional statistical approaches, ML algorithms can identify complex and non-linear relationships among multiple predictors and generate individualized risk estimates using large, multidimensional datasets (9). ML-based prediction models can incorporate routinely collected demographic, anthropometric, lifestyle, clinical, and electronic health record (EHR)-derived data to identify individuals at increased risk of T2DM and support early risk stratification in primary care (8, 9). As AI-enabled clinical decision support becomes increasingly integrated into primary care, ML-based models have the potential to assist healthcare professionals in identifying high-risk individuals, prioritizing confirmatory diagnostic testing, and facilitating timely preventive interventions within routine clinical workflows (10, 11).

Primary care is the first point of contact for most individuals within the healthcare system and plays a pivotal role in the prevention, early detection, and long-term management of type 2 diabetes mellitus (T2DM). Risk prediction models intended for use in primary care should therefore be developed and evaluated within this setting, as patient populations, disease prevalence, routinely available predictors, clinical workflows, and intended end-users differ substantially from those in secondary or tertiary care. Consequently, prediction models developed in hospital-based populations may not perform similarly when applied to primary care without appropriate external validation (12, 13). Furthermore, ML-based prediction models designed for primary care can leverage routinely collected electronic health record data and other information available during routine consultations to facilitate opportunistic risk assessment (11). These considerations highlight the importance of evaluating ML-based T2DM risk prediction models specifically developed, validated, or intended for implementation in primary care settings.

Although several reviews have examined artificial intelligence (AI)-based and machine learning (ML)-based approaches for diabetes risk prediction, they have primarily focused on model performance, algorithm comparisons, or mixed healthcare settings (8–11). Evidence specific to ML-based T2DM risk prediction models developed, validated, or intended for primary care remains limited. Given the diversity of modeling approaches, predictors, validation strategies, and reported performance measures, a scoping review was considered appropriate to systematically map and characterize the available evidence rather than quantitatively compare model performance. Therefore, this scoping review aimed to map the available evidence on ML-based T2DM risk prediction models in primary care, including model characteristics, predictor variables, modeling approaches, validation methods, and reported performance measures.

## Methodology

This project was pre-registered on the Open Science Framework (OSF) (https://osf.io/mbfrz), and the registration is publicly accessible. The review was conducted according to the Joanna Briggs Institute (JBI) methodology for scoping reviews and is reported in accordance with the Preferred Reporting Items for Systematic Reviews and Meta-Analyses extension for Scoping Reviews (PRISMA-ScR). The review focused on machine learning (ML)-based risk prediction models for type 2 diabetes mellitus (T2DM) in primary care, with particular emphasis on model characteristics, predictor variables, modeling approaches, validation methods, and reported performance measures. The completed PRISMA-ScR checklist is provided as Supplementary material.

Protocol concordance and deviations: The review methods were compared with the registered protocol. The final review searched PubMed/MEDLINE, Scopus, and Ovid MEDLINE for studies published between January 2011 and December 2025. These database and publication-period elements remained unchanged from the registered protocol. Title and abstract screening and full-text assessment were conducted independently by two reviewers, with disagreements resolved through discussion and consensus. The registered protocol did not explicitly specify the number of reviewers involved in study selection; therefore, the involvement of two independent reviewers represents a procedural deviation from the registered protocol and is transparently reported here.

### Eligibility criteria

The eligibility criteria were developed using the Population–Concept–Context (PCC) framework recommended by the Joanna Briggs Institute for scoping reviews.

#### Population

- Studies were eligible if they included adults aged 18 years or older from the general population or individuals without established type 2 diabetes undergoing risk assessment early detection, case-finding, or prediction of T2DM.
- Studies conducted exclusively among children or adolescents were excluded because diabetes risk prediction models and screening approaches differ substantially across age groups.

#### Concept

Studies were included if they described machine learning (ML)-based or artificial intelligence (AI)-based models developed for risk prediction, early detection, case-finding, or estimation of the probability of type 2 diabetes in primary care populations.

- Eligible studies included model development, external validation, model updating, comparative modeling, or implementation studies involving ML or AI approaches, including models developed using electronic health records (EHRs) or other routinely collected clinical data.
- Both prognostic risk prediction models (estimating future risk of developing T2DM) and diagnostic/case-finding models (identifying previously undiagnosed prediabetes or diabetes) were considered eligible.
- Studies focusing solely on laboratory-based diagnostic tests (e.g., HbA1c, fasting plasma glucose, or oral glucose tolerance tests) without a predictive modeling component, non-predictive digital interventions, or non-digital risk assessment tools were excluded.

#### Context

- Studies were included if the prediction models were developed, externally validated, evaluated, or intended for implementation in primary care or first-contact healthcare settings, including general practice, family medicine, primary healthcare centers, and community-based primary care clinics.
- Studies conducted exclusively in secondary or tertiary care hospitals, specialist clinics, or inpatient settings were excluded unless the model was specifically intended for primary care use.

#### Types of sources of evidence

Primary research studies of any design were eligible for inclusion, including ML model development, external validation, model updating, comparative modeling, and implementation studies. Reviews, systematic reviews, scoping reviews, editorials, commentaries, conference abstracts without full text, study protocols, opinion pieces, and letters were excluded, as the aim of this review was to map original evidence on ML-based risk prediction models for type 2 diabetes in primary care.

Only peer-reviewed full-text articles published in English between January 2011 and December 2025 were included. Gray literature and preprints were excluded.

### Search strategy

An initial search was conducted in PubMed (MEDLINE) to identify relevant keywords and index terms related to ML-based risk prediction models for type 2 diabetes in primary care settings. The titles and abstracts of relevant articles, along with the index terms used to describe them, were examined to identify commonly used search terms. Based on this preliminary search, a comprehensive search strategy was developed using a combination of controlled vocabulary terms (e.g., Medical Subject Headings [MeSH]) and free-text keywords.

The search strategy was developed using the Population–Concept–Context (PCC) framework and was structured around four key concepts: (1) type 2 diabetes, (2) machine learning and artificial intelligence, (3) risk prediction or risk assessment, and (4) primary care. Controlled vocabulary terms (e.g., MeSH) and free-text keywords were combined using Boolean operators (“AND” and “OR”), truncation, and phrase searching where appropriate. The PubMed search strategy was adapted for the remaining databases.

Three electronic databases (PubMed/MEDLINE, Scopus, and Ovid MEDLINE) were searched. Searches were limited to English-language studies published between January 2011 and December 2025. The search period commenced in January 2011 to ensure comprehensive capture of early applications of machine learning methods in clinical prediction research and primary care settings, including studies published before the rapid expansion of ML-based healthcare research after 2015. All database searches were conducted in January 2026.

Records retrieved from each database were exported into Mendeley Reference Manager, where duplicate records were identified and removed prior to screening. The reference lists of all included studies were manually screened to identify additional eligible studies. Gray literature and preprints were not searched. The complete search strategy is presented in Table 1.

**Table 1:** Search strategy.

Search	Query	Records retrieved
PubMed		
#1	((((type 2 diabetes[MeSH Terms]) OR (type 2 diabetes mellitus[MeSH Terms])) OR (T2DM))) OR (diabetes mellitus[MeSH Terms])	583,420
#2	(((((“machine learning”[MeSH Terms]) OR (deep learning[MeSH Terms])) OR (artificial intelligence[MeSH Terms])) OR (machine learning))) OR (artificial intelligence)	477,352
#3	#1 AND #2	5,493
#4	(((((risk prediction) OR (risk model)) OR (prediction model)) OR (predictive model)) OR (risk score)) OR (prognostic model)	3,327,838
#5	#1 AND #2 AND #4	2,705
#6	(((primary health care[MeSH Terms]) OR (family practice[MeSH Terms])) OR (general practice[MeSH Terms])) OR (primary care)	794,610
#7	#1 AND #2 AND #4 AND #6	175
#8	Filters applied: English, Adult: 19+ years, Exclude preprints, from 2011–2025	97
Scopus		
#1	TITLE-ABS-KEY ((“type 2 diabetes” OR “type 2 diabetes mellitus” OR T2DM OR “diabetes mellitus”) AND (“machine learning” OR “deep learning” OR “artificial intelligence”) AND (“risk prediction” OR “risk model” OR “prediction model” OR “predictive model” OR “risk score” OR “prognostic model”) AND (“primary care” OR “family practice” OR “general practice” OR “primary health care”)) AND (LIMIT-TO (LANGUAGE, “English”)) AND (LIMIT-TO (DOCTYPE, “ar”)) AND PUBYEAR > 2012 AND PUBYEAR < 2026	85
Ovid MEDLINE		
#1	(exp Diabetes Mellitus, Type 2/or type 2 diabetes.mp. or type 2 diabetes mellitus.mp. or T2DM.mp.) and (exp Machine Learning/or exp Artificial Intelligence/or exp Deep Learning/or machine learning.mp. or artificial intelligence.mp. or deep learning.mp.) and (risk prediction or risk model or prediction model or predictive model or risk score or prognostic model).mp. and (exp Primary Health Care/or exp Family Practice/or exp General Practice/or primary care.mp.)	12
#2	limit 1 to (english language and yr=“2011 - 2025” and “all adult (19 plus years)” and “remove preprint records”)	4

### Study selection

Following the database searches, all identified citations from PubMed, Scopus, and Ovid were exported and compiled in Mendeley reference management software. Duplicate records were identified and removed.

Titles and abstracts were independently screened by two reviewers. Potentially eligible articles were subsequently assessed independently by the same two reviewers through full-text review. Disagreements at any stage were resolved through discussion and consensus. The registered protocol did not specify the number of reviewers involved in the screening process; therefore, the use of two independent reviewers represents a procedural deviation from the registered protocol. The database selection and publication period remained consistent with the registered protocol.

### Data extraction

Data were extracted from the included studies using a structured data-charting form developed specifically for this review. The charted information included the first author, year of publication, country, study design, data source, sample size, type of machine learning model, predictors included, predictor selection methods, modeling approach, validation strategy, reported performance measures (e.g., area under the receiver operating characteristic curve [AUC], sensitivity, specificity, accuracy, calibration), and key findings relevant to ML-based risk prediction of type 2 diabetes in primary care.

The data-charting form was developed in accordance with the review objectives and refined iteratively during the extraction process to ensure that all relevant information was consistently captured. Data extraction was performed using the standardized charting form and independently verified by a second reviewer for completeness and accuracy. Any discrepancies identified during the verification process were resolved through discussion and consensus. The extracted information was subsequently organized in tabular format to facilitate comparison across studies.

### Data items

Data were sought on publication and study characteristics, including author and year of publication, country, study objective, study design, data source, sample size, and study population and setting. Information on prediction models included predictor type, prediction objective, ML algorithm(s) or model, predictors included, predictor selection criteria, validation approach, and reported model performance measures, including AUC, sensitivity, specificity, accuracy, F1-score, precision, recall, and calibration, where available. Key findings and research gaps identified by the authors were also charted. Where multiple eligible models were reported within a study, relevant information was recorded for each model. Terminology and performance measures were retained as reported in the original studies, and no assumptions or statistical transformations were applied to unavailable or incompletely reported data.

### Synthesis of results

The findings were synthesized descriptively using both tabular and narrative approaches. The included studies were mapped according to publication characteristics, study setting, data sources, predictor domains, machine learning algorithms, validation approaches, and reported model performance. A narrative synthesis was undertaken to identify and summarize patterns, similarities, and variations in the development, validation, and application of ML-based risk prediction models for type 2 diabetes in primary care. As this was a scoping review, no formal quality appraisal or quantitative meta-analysis was performed.

## Results

### Study/source of evidence inclusion

The database search identified 186 records, including 97 from PubMed, 85 from Scopus, and 4 from Ovid. After removing 21 duplicate records, 165 unique records remained for screening.

Titles and abstracts were screened against the predefined eligibility criteria, resulting in the exclusion of 155 records. A total of 10 articles were retrieved for full-text assessment.

Following full-text review, 6 articles were excluded for failing to meet the inclusion criteria. Four studies met the eligibility criteria and were included in the final scoping review.

**Figure 1:**
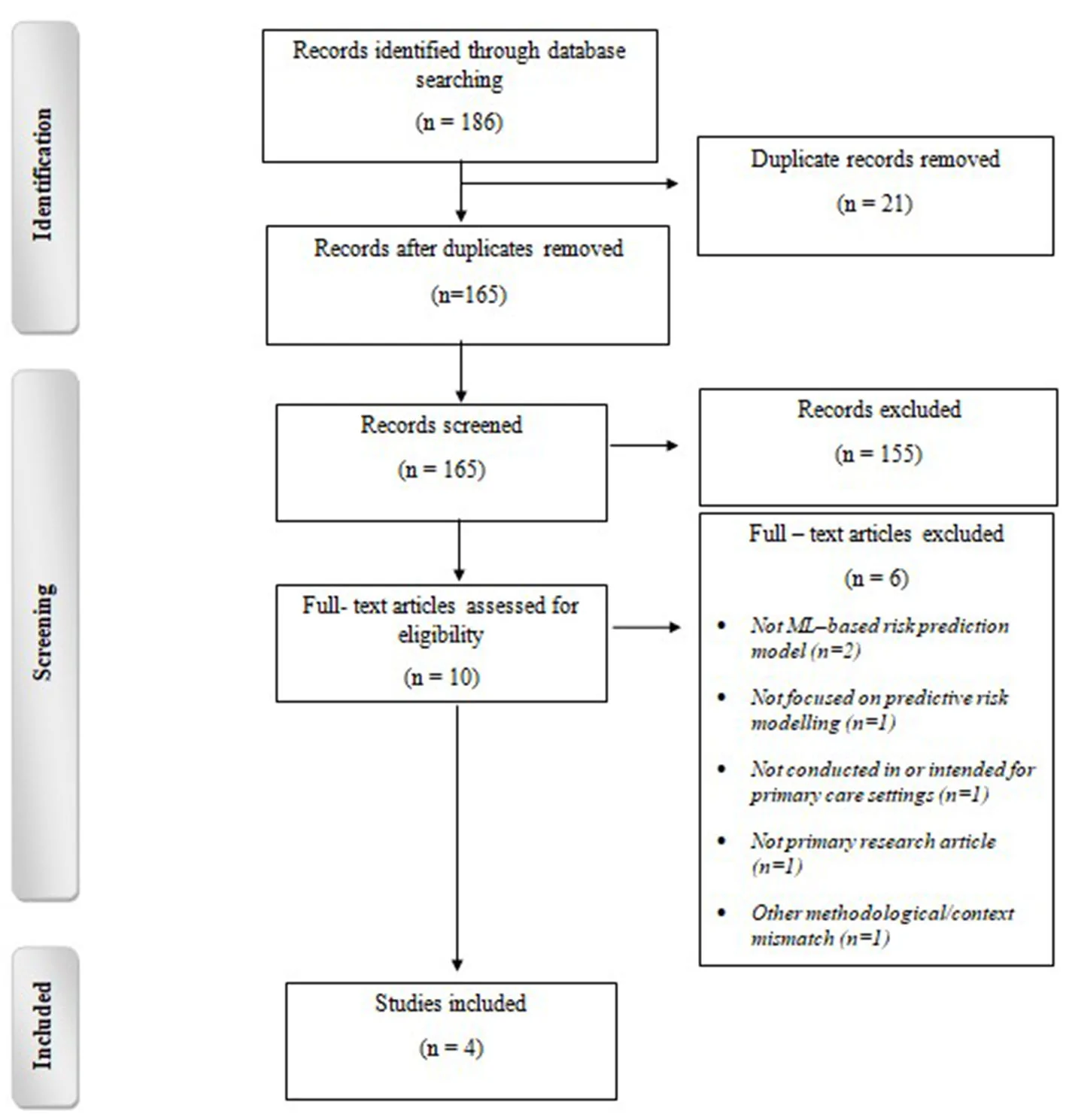
PRISMA-ScR flow diagram of the study selection process.

The study selection process is presented in Figure 1.

### Characteristics of included sources

The characteristics of the included studies are summarized in Table 2. The four studies were published between 2024 and 2025 and were conducted in Sweden, Canada, Hong Kong, and Saudi Arabia (14–17). The studies included retrospective observational, retrospective cohort, observational analytical, and cross-sectional external validation designs. All studies focused on the development, evaluation, or validation of machine learning (ML)-based approaches for risk prediction, early detection, or case-finding of type 2 diabetes in primary care settings. Two studies primarily focused on predicting future diabetes risk (14, 15), whereas two studies focused on identifying previously undiagnosed diabetes or prediabetes within primary care populations (16, 17). Three studies developed ML-based models using routinely collected primary care or population-based datasets (14–16). whereas one study externally validated previously developed non-laboratory diabetes risk prediction models in an independent primary care population (17). Data sources included electronic health records, national primary care databases, questionnaire-based assessments, and population health datasets, highlighting the increasing use of routinely collected clinical information for diabetes risk prediction and case-finding in primary care. Study-specific research gaps identified from the included sources are also presented in Table 2.

**Table 2:** Design and population characteristics of included studies (*N* = 4).

References	Country	Study objective	Study design	Data source	Sample size	Study population/setting	Predictor type	Research gap identified
Wändell et al. (16)	Sweden	To develop a machine learning tool for identifying newly diagnosed type 2 diabetes in primary care	Retrospective observational study	Primary care electronic health records	N = 311,232	Adults attending primary care centers in Sweden	EHR-derived clinical	The model was based primarily on primary-care diagnostic and consultation data; future research should assess whether incorporating lifestyle factors, laboratory tests, and prescribed medication improves prediction performance.
Tang et al. (15)	Canada	To develop and validate federated machine learning models for diabetes prediction across multiple provinces	Retrospective cohort study	Canadian Primary Care Sentinel Surveillance Network (CPCSSN) electronic health records	N = 11,631	Adults receiving care in primary care practices across Canadian provinces	EHR-derived clinical	The study evaluated only logistic regression and MLP and did not assess model calibration; future research should evaluate a wider range of ML algorithms and incorporate calibration in federated diabetes prediction.
Alkattan et al. (14)	Sauvdi Arabia	To evaluate the utility of a machine learning model for identifying individuals at high risk of type 2 diabetes	Observational analytical study	Population-level clinical dataset	N = 3,400 selected; *n* = 679 included in final validation analysis	Adults eligible for diabetes risk assessment relevant to primary care screening	Clinical and demographic predictors (non-laboratory)	The model was externally evaluated against the ADA risk assessment tool rather than actual diagnostic outcomes, highlighting the need for validation against clinically confirmed T2DM outcomes.
Cheng et al. (17)	Hong Kong	To externally validate non-laboratory prediction models for identifying prediabetes and type 2 diabetes in primary care	Cross-sectional external validation study	Questionnaire and clinical assessment data collected in primary care	N = 919	Chinese adults attending public and private primary care clinics	Non-laboratory predictors	The models showed poor calibration in the Hong Kong primary-care population, highlighting the need for recalibration and further validation across diverse populations and primary-care settings.

CPCSSN, Canadian Primary Care Sentinel Surveillance Network; EHR, electronic health record. Predictor type refers to the variables used in the prediction model and does not necessarily reflect the laboratory methods used to ascertain diabetes outcomes.

### Characteristics of machine learning–based prediction models

The characteristics of the ML-based risk prediction models are summarized in Table 3. The included studies differed in their objectives, predictor variables, modeling approaches, validation strategies, and reported performance measures, reflecting the evolving application of ML for diabetes risk prediction in primary care.

**Table 3:** Characteristics of ML-based risk prediction models for type 2 diabetes in primary care (N = 4).

References	Prediction objective	ML Algorithm(s)/ Model	Predictors included	Predictor selection criteria	Validation approach	Performance results	Main findings
Wändell et al. (16)	Case-finding/early detection of previously undiagnosed diabetes	Stochastic Gradient Boosting (compared with logistic regression)	Routinely collected EHR variables including age, sex, hypertension, obesity, lipid disorders, and diagnostic information	Variables derived from routinely collected primary care EHR data and diagnostic codes	Internal validation	AUC: 0.773–0.825; Sensitivity: 0.722–0.744; Specificity: 0.647–0.746	The ML model demonstrated good discrimination for identifying previously undiagnosed diabetes using routinely collected primary care data.
Tang et al. (15)	Risk prediction of type 2 diabetes	Federated Multilayer Perceptron (MLP) and Federated Logistic Regression	Demographic characteristics and routinely collected primary care EHR variables	Predictors selected from routinely collected cross-provincial primary care datasets	Cross-jurisdictional (federated) validation	Performance was assessed using AUC, F1-score, precision, recall, and calibration. Numerical performance values were not consistently reported in the extracted results; federated MLP showed comparable performance to centralized learning, whereas federated logistic regression showed an 8–13% reduction in AUC and 16–20% reduction in F1-score compared with centralized models.	Federated learning enabled diabetes risk prediction across multiple primary care datasets while preserving patient privacy, with good predictive performance across provinces.
Alkattan et al. (14)	Risk prediction of type 2 diabetes	Machine learning prediction model; logistic regression, random forest, linear SVC, decision tree, gradient-boosted tree, and naïve Bayes algorithms were evaluated	Age, sex, BMI, hypertension, smoking status, family history of diabetes, and other clinical risk factors	Clinical and demographic risk factors associated with T2DM risk	Model development and evaluation using the available dataset, followed by evaluation in 679 participants using the selected model	Logistic regression: accuracy 80.88%, recall 76.36%, AUC 0.788 (95% CI: 0.787–0.789). Final ML model evaluation: AUC 0.803 (95% CI: 0.779–0.826), sensitivity 77.94%, specificity 75.13% at cutoff ≥0.5	The final ML model demonstrated good discrimination and classification performance for identifying individuals at high risk of T2DM and showed potential for population-level risk screening in primary care.
Cheng et al. (17)	Case-finding and external validation of prediabetes/diabetes detection models	Previously developed ML model, Logistic Regression model, and simplified risk-scoring algorithm	Age, sex, waist-to-hip ratio, physical activity, vigorous exercise, sleep duration, fruit consumption, family history, and other non-laboratory variables	Predictors included in previously developed non-laboratory diabetes risk models	External validation in an independent primary care population	ML model: AUC 0.744 (95% CI: 0.713–0.773); Sensitivity 69% (95% CI: 65–74%); Specificity 67% (95% CI: 62–71%). Calibration was poor, indicating underestimation of risk.	Both ML and logistic regression models demonstrated satisfactory discrimination (AUC 0.739–0.744) but poor calibration, supporting the need for model recalibration before wider implementation in primary care.

AUC, Area under the receiver operating characteristic curve; BMI, Body mass index; EHR, electronic health record; F1-score, Harmonic mean of precision and recall; ML, machine learning; NPV, Negative predictive value; PPV, Positive predictive value.

#### Study objectives

The included studies addressed two distinct modeling objectives: prognostic risk prediction and diagnostic/case-finding approaches for early detection of undiagnosed disease. Although all four studies aimed to improve the early identification of type 2 diabetes in primary care, their objectives varied. Alkattan et al. (14) evaluated the utility of a machine learning model for identifying individuals at high risk of type 2 diabetes in primary care. Wändell et al. (16) developed a machine learning model using routinely collected primary care diagnostic data to identify newly diagnosed diabetes. Tang et al. (15) proposed a federated machine learning approach that enabled diabetes prediction across multiple Canadian primary care datasets while preserving patient privacy. In contrast, Cheng et al. externally validated previously developed non-laboratory machine learning and logistic regression risk prediction models for identifying prediabetes and diabetes among Chinese adults attending primary care clinics (17).

#### Predictor characteristics

The prediction models incorporated a broad range of predictors spanning demographic, anthropometric, lifestyle, and clinical domains (14–17). Age and sex were consistently included across studies, while anthropometric variables commonly comprised body mass index and waist-to-hip ratio. Lifestyle-related predictors included smoking status, physical activity, sleep duration, dietary habits, and family history of diabetes (14, 17). The included studies used a combination of demographic, anthropometric, lifestyle, laboratory, and EHR-derived clinical predictors, with variation in the availability and type of predictors across models. Three studies additionally utilized routinely collected electronic health record variables, including hypertension, obesity, lipid disorders, diagnostic codes, consultation frequency, and healthcare utilization, enabling automated risk prediction using information already available within primary care systems (14–16). One study externally validated non-laboratory diabetes risk prediction models based on demographic, anthropometric, lifestyle, and family-history variables (17).

#### Model development and validation approaches

A range of ML approaches was identified, including stochastic gradient boosting, federated learning, multilayer perceptron, random forest, support vector classification, naïve Bayes, and decision tree algorithms (14–16). Logistic regression was commonly used as a benchmark comparator or alongside ML models (14, 15, 17). Most studies performed internal validation using cross-validation or cross-jurisdictional validation approaches (14–16), whereas only one study evaluated model performance through external validation in an independent primary care population (17), highlighting the limited evidence regarding the generalisability of current prediction models.

#### Model performance

Model performance was assessed using measures of discrimination, classification, and calibration, including the area under the receiver operating characteristic curve (AUC), sensitivity, specificity, predictive accuracy, F1-score, recall, positive and negative predictive values, and calibration (14–17). Among the studies reporting numerical performance estimates, Wändell et al. reported AUC values ranging from 0.773 to 0.825, with sensitivity ranging from 72.2% to 74.4% and specificity from 64.7% to 74.6%. Alkattan et al. reported an accuracy of 80.88%, recall of 76.36%, and AUC of 0.788 (95% CI: 0.787–0.789) for the logistic regression model during algorithm comparison. In the subsequent evaluation of the final ML model, the AUC was 0.803 (95% CI: 0.779–0.826), with sensitivity of 77.94% and specificity of 75.13% at a cutoff of ≥0.5. Cheng et al. reported an AUC of 0.744 (95% CI: 0.713–0.773), sensitivity of 69% (95% CI: 65–74%), and specificity of 67% (95% CI: 62–71%) for the ML model, with poor calibration indicating underestimation of risk. Tang et al. assessed AUC, F1-score, precision, recall, and calibration; however, numerical values for these measures were not consistently available for comparison in the included report. Federated multilayer perceptron showed performance comparable to centralized learning, whereas federated logistic regression demonstrated an 8–13% reduction in AUC and a 16–20% reduction in F1-score compared with centralized models.

## Discussion

This scoping review identified a limited but emerging body of literature on machine learning (ML)-based risk prediction models for type 2 diabetes mellitus (T2DM) in primary care. Only four studies met the eligibility criteria, highlighting the limited evidence specifically focused on ML models developed, validated, or intended for implementation in primary care. Rather than indicating limited interest in AI-based diabetes prediction, this finding suggests that most published ML prediction studies have been conducted in hospital, specialist, or general population settings, with comparatively little emphasis on first-contact healthcare. Nevertheless, the included studies demonstrate increasing interest in using routinely collected clinical and electronic health record (EHR)-derived data to support earlier identification of individuals at increased risk of T2DM, while also reflecting a gradual shift from model development toward external validation and clinical applicability (14–17). Although model development remains the primary focus of published studies, the inclusion of an external validation study reflects increasing recognition of the importance of evaluating prediction models before routine clinical implementation (12, 13, 18, 19).

Considerable heterogeneity was observed across the included studies with respect to study populations, data sources, predictor selection, modeling techniques, validation strategies, and reported performance measures. Such variation is expected because prediction models were developed within different healthcare systems using diverse routinely collected datasets and were designed for different clinical objectives. Differences in predictor availability, outcome definitions, and validation methods make direct comparison between studies challenging and currently limit the identification of a single optimal prediction approach for primary care. Similar methodological heterogeneity has been highlighted in previous reviews of AI-based diabetes prediction and clinical prediction models, suggesting that standardization of reporting and validation remains an important priority (8–13).

Unlike hospital settings, primary care requires prediction models that rely on routinely available information, are easy to interpret, and can be integrated into routine clinical workflows without increasing the burden on healthcare professionals or patients. Encouragingly, the studies included in this review largely reflected these requirements by incorporating demographic characteristics, anthropometric measures, lifestyle-related variables, and EHR-derived clinical information that are routinely available during primary care consultations (14–17). These findings suggest that ML-based prediction models have the potential to support opportunistic risk assessment, prioritize individuals for confirmatory laboratory testing, and facilitate earlier preventive interventions within routine primary care practice.

An important observation from this review is the variation in the types of predictors used across the included models. Several models relied primarily on non-laboratory variables, including age, sex, body mass index, hypertension, smoking status, lifestyle characteristics, and family history of diabetes. These predictors are consistent with established diabetes risk assessment tools such as FINDRISC, QDiabetes, AUSDRISK, and the Indian Diabetes Risk Score, suggesting that ML approaches can build upon clinically meaningful predictors already used in primary care (20–25). Other models incorporated laboratory measures, EHR-derived clinical variables, or healthcare utilization patterns, highlighting opportunities to use information already available within routine healthcare systems (14–16). This variation reflects differing approaches to risk assessment and the availability of predictors across primary care settings.

Although increasingly sophisticated ML algorithms were evaluated across the included studies, logistic regression remained a commonly used comparator in three of the four studies despite growing interest in AI-based approaches. Previous systematic reviews have similarly reported that ML algorithms do not consistently outperform logistic regression for structured clinical prediction problems when routinely collected clinical variables are used (26–28). Therefore, improvements in predictive accuracy should be considered alongside model interpretability, transparency, feasibility, and ease of implementation when selecting prediction models for primary care.

An important observation from this review is that the included studies addressed two distinct modeling objectives. While Alkattan et al. and Tang et al. focused primarily on predicting an individual's future risk of developing type 2 diabetes, Wändell et al. and Cheng et al. focused on identifying previously undiagnosed diabetes or prediabetes within primary care populations (14–17). Although both approaches aim to facilitate earlier intervention and improve diabetes prevention and management, they represent different modeling tasks with distinct outcomes, target populations, time horizons, and validation requirements. Future studies should clearly distinguish prognostic risk prediction models from diagnostic or case-finding models to improve comparability and interpretation of findings.

Another important finding was the limited evidence regarding external validation and model generalisability. Most included studies relied primarily on internal validation strategies, whereas only one study evaluated previously developed prediction models in an independent primary care population (17). External validation is essential to determine whether prediction models maintain acceptable performance across different populations and healthcare systems before widespread clinical implementation (12, 13, 19). Greater adherence to reporting frameworks such as TRIPOD may further improve transparency, reproducibility, and confidence in future prediction model research (13, 29).

The included studies also highlighted important gaps in the clinical validation and calibration of ML-based prediction models. Alkattan et al. evaluated the developed model against the ADA risk assessment tool rather than clinically confirmed diagnostic outcomes, while Cheng et al. demonstrated poor calibration despite satisfactory discrimination, indicating potential underestimation of predicted risk (14, 17). In addition, Wändell et al. (16) suggested that incorporating lifestyle factors, laboratory measures, and prescribed medication could further assess the potential of ML for diabetes risk prediction. These findings indicate that future studies should evaluate prediction models against clinically relevant outcomes, assess calibration alongside discrimination, and examine whether additional clinically relevant predictors improve model performance and clinical utility.

Despite encouraging predictive performance, evidence regarding real-world implementation remains limited. Most studies focused on retrospective model development or validation rather than prospective evaluation within routine clinical practice. This reflects a broader challenge in AI research, where technically accurate prediction models frequently lack evidence regarding clinical usefulness, workflow integration, clinician acceptance, patient engagement, and long-term sustainability (30–33). Future research should therefore move beyond algorithm development to evaluate implementation outcomes and determine whether ML-based prediction models improve clinical decision-making and patient outcomes in routine primary care.

This review has several strengths. To our knowledge, it is among the first scoping reviews specifically examining ML-based T2DM risk prediction models intended for primary care. The review followed Joanna Briggs Institute methodology, adhered to PRISMA-ScR reporting guidance, and was prospectively registered, thereby enhancing methodological transparency and reproducibility.

Although formal critical appraisal was beyond the scope of this scoping review, reporting practices and completeness of model reporting varied across the included studies. Most studies reported discrimination measures such as AUC, whereas calibration metrics and details necessary for comprehensive assessment were less consistently reported. Future prediction model studies would benefit from adherence to reporting frameworks such as TRIPOD and consideration of PROBAST recommendations to improve transparency, reproducibility, and comparability.

Overall, the findings highlight important priorities for future research. There is a need for externally validated, clinically interpretable, and implementation-oriented ML prediction models that can be applied across diverse healthcare systems and populations. Future studies should place greater emphasis on calibration, clinical impact, cost-effectiveness, and integration into routine primary care workflows using prospective and hybrid effectiveness-implementation study designs (34). Greater representation of low- and middle-income countries is also required to ensure that ML-based prediction models are applicable to settings where the burden of T2DM is increasing most rapidly.

### Limitations

This review has several limitations. First, only peer-reviewed studies published in English were included, and gray literature was excluded, which may have resulted in the omission of relevant evidence. The search was restricted to the databases available through our institution, which may have limited the retrieval of some relevant studies. Second, only four studies met the eligibility criteria, limiting the breadth of evidence available for synthesis. Third, substantial heterogeneity in study populations, data sources, predictor variables, machine learning algorithms, validation methods, and reported performance measures precluded direct comparison across studies. Finally, consistent with the objectives of a scoping review and the Joanna Briggs Institute methodology, no formal critical appraisal or risk-of-bias assessment was undertaken; therefore, the methodological quality of the included studies was not evaluated.

## Conclusion

This scoping review identified a limited but growing body of evidence on ML-based risk prediction models for T2DM in primary care. The included studies demonstrate that ML approaches can utilize routinely available demographic, lifestyle, clinical, and EHR-derived data to support early identification of individuals at increased risk of T2DM. However, evidence remains limited regarding external validation, clinical validation against confirmed outcomes, calibration, and implementation in routine primary care practice. Future research should prioritize externally validated and clinically interpretable prediction models, with greater emphasis on calibration, validation against clinically relevant outcomes, and evaluation of additional predictors where appropriate. Prospective implementation studies should assess clinical utility, cost-effectiveness, and integration into routine primary care workflows. Strengthening the evidence base in low- and middle-income countries will also be essential to support equitable implementation of ML-based diabetes risk prediction in settings with a high and growing burden of T2DM.
